# Which neural mechanisms mediate the effects of a parenting intervention program on parenting behavior: design of a randomized controlled trial

**DOI:** 10.1186/s40359-017-0177-0

**Published:** 2017-03-21

**Authors:** Laura Kolijn, Saskia Euser, Bianca G. van den Bulk, Renske Huffmeijer, Marinus H. van IJzendoorn, Marian J. Bakermans-Kranenburg

**Affiliations:** 1 Centre for Child and Family Studies, Leiden University, P.O. Box 9555, Leiden, 2300 RB Netherlands; 2 Leiden Consortium on Individual Development, Leiden University, P.O. Box 9555, Leiden, 2300 RB Netherlands; 3 Leiden Institute for Brain and Cognition, P.O. Box 9600, Leiden, 2300 RC Netherlands

**Keywords:** EEG, Parenting, Intervention, Emotion, Inhibitory control

## Abstract

**Background:**

The Video-feedback Intervention to promote Positive Parenting and Sensitive Discipline (VIPP-SD) has proven effective in increasing parental sensitivity. However, the mechanisms involved are largely unknown. In a randomized controlled trial we examine parental neurocognitive factors that may mediate the intervention effects on parenting behavior. Our aims are to (1) examine whether the intervention influences parents’ neural processing of children’s emotional expressions and the neural precursors of response inhibition and to (2) test whether neural changes mediate intervention effects on parenting behavior.

**Methods:**

We will test 100 mothers of 4–6 year old same-sex twins. A random half of the mothers will receive the VIPP-SD Twins (i.e. VIPP-SD adapted for twin families), consisting of 5 home visits in a 3-months period; the other half will receive a dummy intervention. Neurocognitive measures are acquired approximately 2 weeks before and 2 weeks after the intervention. Mothers’ electroencephalographic (EEG) activity is measured while performing a stop signal task and in response to children’s facial expressions. To obtain a complementary behavioral measure, mothers also perform an emotion recognition task. Parenting behavior will be assessed during parent–child interactions at pre and post intervention lab visits.

**Discussion:**

Our results will shed light on the neurocognitive factors underlying changes in parenting behavior after a parenting support program, which may benefit the development of such programs.

**Trial registration:**

Dutch Trial Register: NTR5312; Date registered: January 3, 2017.

**Electronic supplementary material:**

The online version of this article (doi:10.1186/s40359-017-0177-0) contains supplementary material, which is available to authorized users.

## Background

Parents play a pivotal role in children’s social, emotional and cognitive development (e.g., [1, 2]). Parental sensitivity, defined as the ability to recognize, accurately interpret and promptly respond to children’s cues [3], is a core construct indicating quality of parenting. Parental sensitivity has been found to be an important predictor of children’s internalizing and externalizing problem behavior [4–7], social competence [8, 9] and emotion regulation [10, 11]. The Video-feedback Intervention to promote Positive Parenting and Sensitive Discipline (VIPP-SD) [12] has been proven to enhance parental sensitivity and sensitive discipline in several randomized controlled trials in various countries [13]. However, the underlying mechanisms accounting for the observed change in parenting behavior remain largely unknown. The current protocol presents a randomized controlled trial in which we aim to examine the neurocognitive mechanisms through which intervention effects on parenting behavior might be established. The focus will be on assessing the underlying neural activity of two constructs that may be important in parenting behavior: emotion recognition and inhibitory control.

### Parental sensitivity

To promote survival, infants are biologically predisposed to develop an attachment relationship with their caregiver [14]. A secure attachment relationship is established through early caregiving experiences and is related to positive outcomes in early and later childhood and adolescence [15–18], highlighting the importance of developing a secure attachment relationship. More specifically, meta-analytic studies confirm that insecure and disorganized attachment is related to later externalizing problem behavior [19], internalizing symptoms [20] and poorer social competence [21]. An important determinant for developing a secure attachment relationship is parental sensitivity [22, 23], as changes in parental sensitivity have been shown to lead to changes in attachment security in children [23]. Enhancing parental sensitivity thus benefits the quality of the attachment relationship which in turn is supposed to lead to positive child outcomes [4–7, 24, 25].

Although early caregiving experiences during infancy and early childhood are central to developing a secure attachment relationship, parents’ responses to their children’s communications regarding feelings of anxiety and stress remain of great importance during childhood. Neuropsychological research into parenting provides insight into parents’ processing of and responding to children’s attachment cues. For example, EEG research can provide insight into which specific early, automatic processes (e.g. face perception) and/or later, more controlled (‘reflective’) processes (e.g. resource allocation [26]) contribute to (successful and sensitive) parental behavior. Outcomes may have implications for the malleability of parental responses as well as the kind of interventions needed to optimize parental sensitivity. We will investigate neural processing of emotional facial expressions and the neural correlates of inhibitory control as it is plausible that these processes are important for parental sensitivity. More specifically, the two neurocognitive processes of interest may be affected by the intervention since key elements of the intervention involve parental coping with children’s displays of (negative) emotionality.

### Processing facial expressions

An important aspect of parenting is recognition and accurate interpretation of emotional child cues, for example emotional facial expressions. An extensive body of EEG research on faces reports the N170 to be a neurophysiological marker of face processing. The N170 is a negative-going event-related potential (ERP) component that peaks at approximately 170 ms post stimulus onset at occipito-temporal electrode sites and is usually largest over the right hemisphere. The N170 is thought to reflect the relatively early stage of processing and encoding face configuration (e.g., [27, 28]) (for a recent review see [29]). Although there is some debate regarding effects of emotional valence on N170 amplitude and latency (with contradictory findings; [30–35]), N170 amplitudes are generally larger for emotional compared to neutral faces (see [36], for a meta-analysis) and there is evidence that N170 amplitude is sensitive to the intensity of emotional expressions [34]. In addition, individual differences in socio-emotional characteristics (e.g., [37–39]) as well as negative childhood parenting experiences [40, 41] have been found to affect N170 and VPP amplitudes (thought to reflect activity of the same set of generator dipoles; [42]). Importantly, a recent study has provided initial evidence that the neural processing of children’s emotional facial expressions may be responsive to behavioral intervention: Neural activity in response to emotional facial expressions was found to be different in Child Protective Services (CPS)-referred mothers who received an attachment-based intervention compared to a randomized control group [43]. In the current study we aim to test whether the intervention will affect the N170 in response to children’ emotional faces in a large non-clinical sample of mothers of young same-sex twins. To complement neural data on processing facial expressions, mothers will perform an Emotion Recognition Task (ERT; [44]) to measure facial emotional processing at the behavioral level. The ERT measures perception of facial emotional expressions presented at different intensities. The ERT contains neutral child faces (0% emotional expression) that gradually (i.e. in 10% steps) change into an emotional expression (100% emotional expression). By pressing a button, mothers indicate that they recognize the emotion they think is expressed on the face and subsequently select the corresponding emotion they recognized.

### Inhibitory control

Inhibitory control plays a crucial role in emotion regulation [45] and both processes impact parenting behavior, especially in stressful situations [46]. Challenging child behavior may evoke negative parenting, including the use of harsh discipline, and lack of support and structure [47]. Low cognitive control in general has been related to a variety of negative parenting behaviors, such as ineffective and controlling parenting styles, negative reactions toward children’s emotions, maternal rejection and risk for maltreatment [48]. Thus, parents’ efficient control as reflected in the ability to inhibit negative parenting responses to child attachment signals may facilitate parental sensitivity and sensitive discipline when parents are faced with challenging child behavior. In addition, the association between low inhibitory cognitive control and increased negative parenting was found to be stable in parents with children in early childhood through adolescence [48], highlighting the importance of supporting inhibitory capacities in the early stages of parenting.

The amplitude of the N2 component elicited in stop signal tasks (which requires inhibition of a prepotent response at the presentation of a specific stimulus; see [49]) is implicated in inhibitory control over responses (for a review, see [50]). The N2 is a negative-going ERP component that peaks at around 200 ms after stimulus onset at fronto-central electrode sites. The N2 has been found to be involved in response inhibition, and may be affected by a combination of stop signal processing, conflict detection and suppression of motor responses [50–52]. Smaller (less negative) N2 amplitudes have been related to less efficient response inhibition [53] as well as impulsive-violent behavior [54]. As inhibitory control plays an important role in emotion regulation and thereby modulates parental reactions to children’s behavior [46], we aim to test whether the intervention enhances N2 amplitudes as well as the efficiency of response inhibition in a stop signal paradigm.

### Parental stress

Parenting behavior can be negatively influenced by parental stress [55, 56]. For example, parents who experience more daily stressors show more lax and harsh parenting behavior, and may lack warmth and responsiveness [57, 58] and daily hassles influence both parenting behavior and parent–child interactions [59]. Parenting interventions may be effective in enhancing parental feelings of efficacy, and in reducing reported parental stress [60]. Stressful life events are robustly related to heightened cortisol levels, and in a previous study a parenting intervention was found to be effective in reducing cortisol levels in children carrying the DRD4 7-repeat allele [61]. For the current study we aim to investigate whether the intervention lowers stress in parents, as reflected in self-reported stress and in lower cortisol levels, which in turn may facilitate parental sensitivity.

### Intervention

The VIPP-SD aims to enhance parental sensitivity and sensitive discipline [12] and has been proven to be effective in twelve randomized controlled trials in various populations (combined effect size of d = 0.47 [13]). For the current study, the VIPP-SD protocol was adapted for families with young same-sex twin children, the VIPP- SD Twins [62]. Compared to parents of singletons, parents of twins are exposed to more parenting challenges that may put them at risk for developing mental health issues [63]. In addition, parents of twins experience more parenting stress and depression, experience parenting as more difficult and obtain less pleasure from their children [64], highlighting the importance of parenting support for twin families.

### Aims and hypotheses

**Fig. 1:**
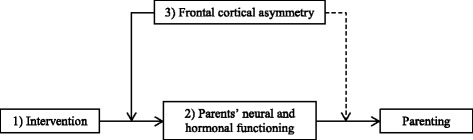
Overview of central study parameters and aims. Note. The numbers in the figure correspond to the order of the aims of the study

1. Our primary aim is to investigate intervention effects on the neural correlates of inhibitory control and the neural processing of emotional facial expressions. First, we will examine whether the intervention affects the neural processing of children’s emotional faces as reflected in the N170 component. We expect that N170 amplitudes in response to emotional faces will be enhanced in parents in the intervention condition compared to parents in the control condition. In addition, we will explore potential latency and differential emotion effects as well. Second, we will examine whether the intervention affects the N2 during a response inhibition (stop signal) task. Compared to parents in the control condition, we expect N2 amplitudes in response to stop signals to be enhanced in parents in the intervention condition. In addition, we will explore whether the intervention affects latency of the N2.
2. Our secondary aim is to investigate the neurobiological mechanisms through which intervention effects on parenting behavior are established. More specifically, we will investigate whether the intervention results in changes in these neurocognitive processes which in turn contribute to observable effects on parenting behavior. We will examine whether intervention effects on parenting behavior are mediated by intervention effects on the N170 and N2. The expectation is that the intervention positively affects the neural processing of children’s emotional faces and inhibitory control mechanisms, as indicated by enhances amplitudes of the N170 and the N2, which in turn will promote parental sensitivity and sensitive discipline during parent–child interactions. In addition, we will examine whether intervention effects on sensitive parenting behavior are mediated by the stress hormone cortisol. It is expected that the intervention reduces stress levels in parents which in turn promotes parental sensitivity and sensitive discipline.
3. Our tertiary aim is to explore whether intervention effects on parenting behavior and on N170 and N2 amplitudes are moderated by patterns of asymmetric frontal cortical activity (see Fig. 1). Asymmetric frontal cortical activity is thought to reflect an individual’s motivational tendency toward approach or withdrawal [65]. Individual differences in motivational tendencies may affect their sensitivity to interventions targeting social behavior. In a recent study, for example, we found that effects of administered oxytocin and experiences of love withdrawal on donations to charity were moderated by individual differences in asymmetric frontal cortical activity. Oxytocin and love withdrawal affected donations only for individuals showing greater activity of the right than the left frontal cortex [66]. We expect frontal cortical asymmetry to play a similar moderating role in intervention effects on the N170 and N2, and, ultimately, parenting behavior (Fig. 1).

## Methods/design

### Study design

The current study is part of the Leiden Consortium on Individual Development (L-CID) which is a 5-years randomized controlled trial including a parenting intervention in which families with young same-sex twins living in the western region of the Netherlands participate (for a more detailed description on the full L-CID study design, see [62]). The current study focuses on factors involved in the intervention, with the primary caregiver of the twins as participants. The intervention is delivered to a random 50% of the primary caregivers. The study consists of two assessments in which only the primary caregiver will take part. The first assessment (i.e. pretest) will take place 2 weeks before and the second assessment (i.e. posttest) 2 weeks after the intervention. Both assessments will take place in the laboratory and focus on the neural mechanisms through which intervention effects on parenting behavior are brought about. To measure parenting behavior, parental sensitivity and sensitive discipline will be assessed during the first posttest of the L-CID study in which both the primary caregiver and children take part [62]. This protocol paper adheres to the SPIRIT guidelines (See Additional file 1).

### Participants

#### Recruitment

As the current study is part of the larger L-CID study, recruitment has been completed. Families with twins living in the western region of the Netherlands were selected from municipality records. Families were eligible for participation when twins were same gender, when the parents were fluent in Dutch and when the grandparents were born in Europe (for more detailed information on recruitment, see [62]). For the current study, parents will be excluded in case of a history of or current neurological disorders and/or damage, psychiatric disorders and/or use of psychoactive medication. Parents will be invited for the first assessment by phone after which they will receive a detailed information letter. Parents will receive a financial reimbursement of €20 for participating in each assessment and their travel- and babysitting expenses will be covered.

#### Randomization

Randomization to intervention condition is done every month at the family level in a ratio of 2:3, using a computer-generated blocked randomization sequence, with a block size of 19 families based on timing of the intervention and stratified by gender of the primary parent and twin. For the current study, we will use a condition ratio of 1:1, leading to a group of 50 intervention and 50 control parents. To select this subsample, a similar number of families from the intervention and control condition will be invited for the study, using the same blocked computer-generated randomization sequence and stratified by twin gender, but excluding male primary parents. The remaining families in both the intervention and control condition will be assigned to the intervention or control “shadow sample”. The shadow samples will be used when parents who are assigned to the parent study refuse to participate in this part of the project.

**Fig. 2:**
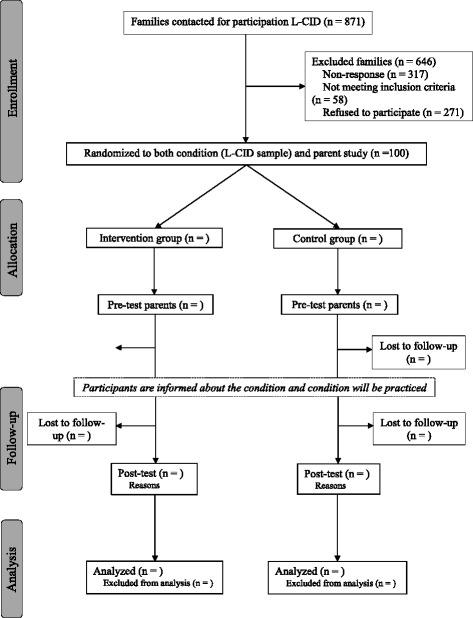
Flowchart of the phases of the randomized-controlled trial

An independent researcher who is not involved in data collection or coding will perform assignment of participants. Right before the start of the intervention, allocation will be performed in order to prevent selective attrition. Because of the open-label design researchers, interveners and participants are blinded to assignment before, but not after, randomization. Importantly, only after the first (pretest) parent assessment has taken place, parents will be informed about the condition they are assigned to (see Fig. 2). Coders and research assistants who carry out the post-intervention home-visits and laboratory sessions are blind to treatment allocation to reduce bias generated by knowledge about allocation of participants to a minimum.

#### Sample size and power

For our primary aim, testing the effect of the intervention on the N170 and N2, with a repeated measures analyses with α = .05 and a sample size of 100 parents, the power to detect at least a medium-sized effect is > .9 (repeated measures ANOVA within-between interaction, G*Power 3.1.9.2). For our secondary aim, testing mediating mechanisms, the power to detect medium to large effects is at least .9 as the power to detect mediating effects is generally larger than it is for main effects [67]. For our third aim, testing moderation effects, the power is to detect medium to large effects is .5–.9.

### Intervention

#### VIPP-SD Twins

The original version of the intervention (VIPP-SD) has been adapted for the use with twin families, the VIPP-SD Twins (see, [62]). Instead of only including one target child in the intervention sessions, both twins are included. Parenting a twin may lead to different kinds of challenges for parents, such as dividing attention and sharing or competition between twins, which are less relevant for parents with singletons (for a detailed description of the adaptions, see [62]). The experimental group (50% of the parent sample, randomly selected) will receive the VIPP- SD Twins between the pre and posttest (see [62, 68] for a detailed description). The VIPP-SD Twins consists of five home visits in which families are visited at home by a female intervener. All interveners were extensively trained and used the manual VIPP-SD version 3.0 [68] that was adapted for twin families [62]. The manual describes the structure, themes, tips, and exercises for parent and children for each session. Every session starts with videotaping approximately 15 min of standardized parent–child interactions, such as playing or reading a book together [69]. Between sessions, the intervener prepares comments on the child’s or parent’s behavior based on the theme of the next session and selects illustrating video fragments. In the next session, after new video material is collected, the intervener reviews the video of the previous session with the parent and gives video feedback on the selected video fragments. The focus of this feedback period, is on positive and successful interaction moments and the intervener indicates when positive parenting is effective. The parent is explicitly acknowledged as the expert on her own child. The first four intervention sessions each have their own themes with respect to sensitivity and sensitive discipline [12]. Subsequently, the four themes focus on exploration versus attachment behavior, perception of the child’s signals, the importance of prompt and adequate responding to child’s signals and sharing emotions. The final session is a booster session, in which the previous themes are repeated and integrated. The parents’ partner is invited to participate in the final session (for details, see [62, 68]).

#### Control condition

To ensure the same number of contact for all participating families, a control condition is implemented. During the same period as the intervention sessions, a research assistant will make six phone calls to families in the control condition. The subject of phone calls will be general development of the twins in a semi-structured interview format. However, families do not receive any specific information or advice about parenting or child development (e.g., [69]).

### Measures

#### Primary aim

Our primary aim is to investigate intervention effects on two neurocognitive processes. First, we will examine whether the intervention affects the neural processing of children’s emotional faces as reflected in the N170, an ERP component reflecting face processing [27]. The N170 will be quantified from participants’ electroencephalographic (EEG) activity recorded during a face processing paradigm. Participants’ EEG will be acquired using 129-channel hydrocel geodesic sensor nets with the NetAmps300 amplifier and NetStation software (Electrical Geodesics, Inc.; EGI). While their EEG is recorded, participants will view pictures of children’s faces with a happy, angry or neutral expression. Pictures were selected from the Child Affective Facial Expression (CAFE) set [70], a validated set of 2- to 8-year-old children’s faces. To make sure that child identity would not vary across emotional categories, we included only pictures of children who had validated pictures for all 3 emotions of interest (*n* = 16 children). During the face processing paradigm, each of the 48 selected faces (i.e. 16 happy, 16 angry and 16 neutral) is presented 3 times in quasi-random order (with the restriction that the same condition cannot occur more than four times in a row), resulting in a total of 144 trials (i.e. 48 happy, 48 angry and 48 neutral). Every trial starts with a fixation cross (duration: 800–1200 ms, varying randomly) followed by the stimulus, that is presented for 1000 ms. Every 24 trials, 10-second blink-breaks were inserted so participants could rest their eyes. In every set of 24 trials (varying randomly between the fifth and twentieth trial), participants are asked about the gender of the child in the previously presented face, to keep participants engaged in the task.

Second, we will examine whether the intervention affects neural activity underlying inhibitory control as reflected in the N2, an ERP component implicated in response inhibition [50]. The N2 will be quantified from participants’ EEG activity recorded (see above) during a stop signal task. During the stop-signal task, participants are presented with a “go”-signal, a green arrow pointing left or right (presented on an black background) that requires a response (pressing the corresponding button on a response pad). On some trials, a “stop”-signal, a red arrow (pointing in the same direction as the preceding green arrow) is presented after the go-signal, and participants should withhold (i.e. inhibit) the response. Every trial starts with a white fixation cross (duration: 800–1200 ms, varying randomly) presented on a black screen followed by a green arrow. In a random 25% of the trials, the go-stimulus is followed by the red arrow. Presentation duration of the green arrow is 15000 ms on go-trials (i.e., no stop-signal is presented) and varies on stop trials depending on the participant’s performance. The duration equals 250 ms at the start of the task and is increased with 50 ms after every successful inhibition and shortened with 50 ms after every unsuccessful inhibition. The task thus becomes more difficult when participants successfully inhibit their responses and less difficult when inhibition is unsuccessful. The stop signal task consists of 400 trials in total, of which 100 are stop-trials.

#### Secondary aim

Our secondary aim is to test if the intervention effects on parenting behavior are mediated by changes in the N170, the N2, and the stress hormone cortisol. To measure cortisol, hair samples (i.e. approximately 100 strands) will be collected during both parent assessments, thus before and after the intervention. Hair strands are collected at the posterior vertex, as close to the scalp as possible (e.g. [71, 72]). Samples are taped to a paper on which the scalp end is marked. The samples are packed in tinfoil and stored at room temperature until analysis. Hair is a valid and non-invasive tool to measure total cortisol release over a longer period of time [72–74] and has been used to determine cortisol levels in both adults and children [71, 75, 76].

Parenting behavior is operationalized as parental sensitivity and sensitive discipline. Parental sensitivity is assessed during free play and structured play situations and discipline is assessed during a compliance task. During the compliance task the parent is asked to instruct the child to do something he or she does not like (e.g., cleaning up or to refrain from touching attractive toys [77, 78]). All parent–child interaction tasks are videotaped and trained coders will code the videos for parental sensitivity and sensitive discipline. For coding purposes, the Erickson 7-point rating scale for Supportive Presence and the 7-point rating scale for Intrusiveness will be used [79]. To prevent coder drift, regular meetings will be organized to discuss videos to obtain intercoder reliability ICC > .65, Pearson’s r > .70. Aggregated measures across ratings and settings will be constructed for each parenting construct.

#### Tertiary aim

For our third aim, we will examine whether intervention effects are moderated by patterns of asymmetric frontal cortical activity. Participants’ EEG activity will be recorded during four periods of ‘rest’: Sitting in a comfortable chair facing a computer screen in a dimly lit room, participants will be asked to “just relax” and keep their eyes focused on a fixation cross (as much as possible) presented on the computer screen. After 2 min, participants are asked to close their eyes for 2 min. This sequence of resting measures will be conducted before starting and after ending of the face processing and stop signal tasks, resulting in 8 min of resting EEG recordings. Differences in power in the EEG alpha band (8–12 Hz) over the left and right frontal cortex (right-left) will be computed to quantify asymmetric frontal cortical activity (e.g., [66]).

#### Statistical analyses

Initial data analysis with data inspection steps will be carried out after the research plan and data collection have been completed but before formal statistical analyses are conducted [80]. We will apply range checks for data values, to check data quality. It will be tested whether missing data are completely at random, at random, or not at random [81], and multiple imputation procedures will be applied to impute missing data. Data transformation will be applied when necessary to approach normal distribution of data points [82]. To avoid any inflation of statistical tests, we are not planning to examine any interim data-sets. For all aims, the effect of the intervention compared to the control condition will be analyzed using intent to treat analyses. For the primary aim, we propose a repeated measures model to estimate the intervention effect on N170 and N2 with experimental condition as between subjects factor and assessment time-point as within subjects factor. The regression coefficient of the interaction between condition and time-point estimates differential neural activity changes between the intervention and control groups over time. For our secondary aim, exploring mechanisms of intervention effects, we will use the Montoya & Hayes approach [83] in a multilevel or repeated measures design to test for intervention effects on neurocognitive variables and examine whether these neurocognitive changes mediate the observed changes in parenting behavior. For our third aim, examining the moderation of the intervention effect, we will include a moderator term in the model.

#### Data management and ethics

Data will be handled strictly confidentially. Data will be stored in the storage environment of the universities Computing Centre in Leiden. Information security is treated in accordance with the International Security Code. Based on European legislation, personal information and data are processed conform the Dutch Personal Information Protection Act and Dutch Personal Data Protection Act. Data and biological specimen is linked to the subject by using a separate subject identification code. Subject are not personally identifiable in scientific communications. Currently, we do not have ethical permission to share data. Only the formal research team, that includes principal investigators, post-docs and PhD-students will have access to the final trial dataset. All research team members signed an agreement of confidentiality.

The L-CID trial is embedded in the larger national Consortium on Individual Development (CID), which unites developmental researchers from seven different universities. CID composed an international scientific advisory board for advice on and supervision of the research program, and a supervisory board to whom our research team reports at least annually.

The research protocol received ethical approval by the Central Committee on Research Involving Human Subjects in the Netherlands (CCMO; NL49069.000.14). An additional informed consent for the current two assessments was obtained before the first assessment, from all participants. Participants were reminded that participating in the trial is voluntary, that their data are stored anonymously and securely and that they can withdraw from the study at any time, without consequences. All consent forms and related documentation given to the participants were approved by the CCMO and can be requested from the authors. Name and contact information of an independent expert (a MD and professor in child and adolescent psychiatry) who will be available during the trial for questions from participants is included in the information for the participants.

The VIPP has been used in twelve previous RCTs, including more vulnerable populations [13, 84]. As there are no reported risks associated with the intervention, there are no criteria for discontinuing the intervention, except on the basis of participants’ own requests (see [62] as well). Concomitant care during the trial is not prohibited, but we will use an inventory about previous or concurrent experiences with video-feedback or other types of preventive care, such as parent training or well-baby clinics. Trial results will be communicated to participants using newsletters about the trial and to professionals in the form of (popular) journal articles and professional or scientific conferences. Authorships for journal articles will be determined based on the APA-guidelines and recommendations from the International Committee of Medical Journal Editors. The trial is registered in the Netherlands Trial Registry (NTR; Trial ID: NRT5312, Date registered: January 3, 2017). Any protocol modifications or plans for ancillary studies will be reported to the NTR, CCMO and this journal, and additional informed consent will be obtained from participants.

## Discussion

The current protocol presents a study design of a randomized controlled trial in which we aim to investigate neural and hormonal mechanisms that may be involved in the intervention effects of the VIPP on parenting behavior. More specifically, we hope to gain insight in the mediating mechanisms through which intervention effects on parenting behavior are brought about. So far, research shows that the VIPP is effective in enhancing parental sensitivity, however the neurocognitive mechanisms involved in enhanced parenting sensitivity remain largely unknown. The results will provide fundamental insight into parenting behavior and intervention efficacy.

### Strengths and limitations

The study has several strengths, such as random assignment to condition, the golden standard to test intervention effects, and the neurobiological and behavioral assessments of mediating, moderating and outcome variables. The VIPP-SD program is firmly rooted in the well-validated attachment theory and social learning theory [12], and has been proven to be effective in enhancing parental sensitivity in a series of randomized controlled trials in several countries [13, 84]). The pretest posttest control group design provides maximum power to trace intervention effects and its mediators.

The study has some limitations as well, such as multiple interveners between families who carry out the intervention. This may introduce variability in intervention efficacy. However, by using a standardized manual and extensive training prior and supervision during the intervention we expect to limit possible intervention divergences. Another possible limitation is that we test parents of twins and therefore the results may be limited in their generalizability.

## Additional file

Additional file 1: SPIRIT Checklist. (DOC 122 kb)

## Abbreviations

EEG: Electroencephalography
ERP: Event-related potential
L-CID: Leiden Consortium on Individual Development
RCT: Randomized Controlled Trial
VIPP- SD Twins: Video-Feedback Intervention to Promote Positive Parenting and Sensitive Discipline in Twin Families
VIPP-SD: Video-Feedback Intervention to Promote Positive Parenting and Sensitive Discipline
